# Cell-Type-Dependent Thyroid Hormone Effects on Glioma Tumor Cell Lines

**DOI:** 10.4061/2011/856050

**Published:** 2011-12-21

**Authors:** Liappas Alexandros, Mourouzis Iordanis, Zisakis Athanasios, Economou Konstantinos, Lea Robert-William, Pantos Constantinos

**Affiliations:** ^1^Department of Pharmacology, University of Athens, 75 Mikras Asias Avenue,11527 Goudi, Athens, Greece; ^2^School of Pharmacy and Biomedical Sciences, University of Central Lancashire, Preston PR1 2HE, Lancashire, UK

## Abstract

*Purpose*. The present study investigated the potential effects of long-term T3 treatment on glioma tumor cell lines. Thyroid hormone action on cell growth, differentiation and survival during development may be of therapeutic relevance *Methods and Results* 1321N1 cell line, an astrocytoma grade II, and U87MG, a glioblastoma grade IV, were exposed for 2 and 4 days in medium deprived of T3 and in medium containing 1 nM T3. T3 promoted re-differentiation in both cell lines. However, T3 increased cell proliferation in 1321N1 (2 days) which declined thereafter (4 days) while in U87MG resulted in suppression of cell proliferation. At the molecular level, a 2.9 fold increase in the expression of TR**α**1 receptor was observed in U87MG versus 1321N1, *P* < 0.05. TR**β**1 receptor was undetectable. These changes corresponded to a distinct pattern of T3-induced kinase signaling activation; T3 had no effect on ERK activation in both cell lines but significantly increased phospho-Akt levels in 1321N1. *Conclusion*. In conclusion, T3 can re-differentiate glioma tumor cells, whereas its effect on cell proliferation appears to be dependent on the type of tumor cell line with aggressive tumors being more sensitive to T3. TR**α**1 receptor may, at least in part, be implicated in this response.

## 1. Introduction

It is now recognized that thyroid hormone (TH) may have a critical role in the pathogenesis and the progression of the diseases due to its regulatory action on cell differentiation, proliferation, and survival [1]. Experimental and clinical studies provide a growing body of evidence that TH signaling may be altered in heart failure with important physiological and therapeutic consequences [2]. Similarly, alterations in TH signaling have been observed in malignancies [3, 4], and hypothyroidism is shown to enhance tumor invasiveness and metastasis development [5, 6]. Furthermore, in 1896, thyroxine (horse thyroid extract) was the first successful hormonal product to be used against a fulminating breast cancer [7]. Similar results were thereafter reported for a series of patients with breast cancer in 1954 [8]. However, until now, the potential of TH as cancer therapy has not been adequately explored.

Gliomas represent the most common primary brain tumor and are among the most aggressive of cancers. Patients with glioma typically relapse within a year of initial diagnosis [9]. Although neurosurgical resection, radiation, and chemotherapy provide clear benefit, prognosis remains disappointing. TH levels are shown to be low in patients with gliomas but the relevance of this response to the pathophysiology of the disease remains largely unknown [10]. However, recent experimental studies provide evidence showing that acute, short-term TH treatment may increase cell proliferation and survival via its nongenomic action [11–13]. In contrast, long-term TH treatment appears to suppress cell proliferation in neuroblastoma cells [5]. Based on this evidence, in the present study, we further explored the long-term T3 effects on glioma tumors in relation to the degree of tumor aggressiveness and potential alterations in thyroid hormone nuclear receptor (TR) expression which may characterize different types of glioma cell lines. This issue although of clinical and therapeutic relevance has not been previously addressed.

## 2. Materials and Methods

### 2.1. Cell Culture

1321N1 cell line, an astrocytoma grade II, and U87MG, a glioblastoma grade IV, were used in this study. Glioma cell line U87MG was obtained from the American Type Culture Collection (ATCC) (Manassas, VA), and glioma cell line 1321N1 was obtained from the European Collection of Cell Culture (ECACC) (Salisbury, Wiltshire, UK). All cell lines were maintained in 150 cm² cell culture flasks (CORNING). U87MG was maintained in Eagle's Essential Minimum Medium (MEM) with Earle's salts supplemented with 10% fetal bovine serum (GIBCO), 1 mM sodium pyruvate, streptomycin and penicillin (5% v/v), 0.1 mM nonessential amino acids, 2 mM L-glutamine, and amphotericin B (5% v/v).

1321N1 was maintained in Dulbecco's Modified Eagle's Medium (DMEM) with glucose and sodium bicarbonate supplemented with 10% FBS, 2 mM L-glutamine, 5% penicillin and streptomycin, and 5% amphotericin B.

All cell lines were maintained in a 37°C humidified incubator with 5% CO_2_. For all experiments, each of the glioma cell lines was used between passages 20–30. Once cells were 70–80% confluent, they were trypsinized using 1X Trypsin. Cells were settled for 24 h in stripped medium (using charcoal FBS, GIBCO) before the initiation of treatment. Cells were cultured for 48 h and 96 h either in stripped medium only (nontreated) or in stripped medium in which 1 nM of T3 was added.

### 2.2. Cell Morphology

Cell morphology was used to assess cell differentiation. Cells were fixed in, before being viewed with an inverted light microscope fitted with phase contrast optics. Five random fields, each containing no more than 50 cells, were examined in each well, and the total number of cells as well as the total number of extensions that were greater than two cell body diameters in length were recorded. Data were derived from approximately 100 cells in each group. Cell morphology could not be reliably assessed at 96 h due to more than 80% confluency in non-treated cells.

### 2.3. Cell Proliferation

In order to measure cell proliferation, BrdU labeling reagent (RPN20 kit, GE Healthcare, Piscataway, NJ) was added to the medium. Cells were incubated for 30 min and then fixed using 4% paraformaldehyde for 15 min. Primary antibody (anti-BrdU monoclonal antibody, dilution 1 : 100) was applied for 1 h at room temperature. Samples were washed 3 × 5 min with PBS. Secondary antibody (peroxidase anti-mouse IgG2a) was then applied for 30 min at room temperature, followed by washing 3 × 5 min. Finally, BrdU-immunostained cultures were visualized using DAB and photographs taken with a digital camera (Zeiss Axiovert) attached to an inverted microscope fitted with phase contrast optics. BrdU-positive nuclei were counted as a percentage of total nuclei. Proliferation data are derived from between 450 to 600 cells measured in each group.

### 2.4. Measurement of Total Cell Number

At 2 and 4 days after treatment, cells were washed twice with PBS, and 100 *μ*L trypsin 0.25% were added to each plate and incubated for 37°C until the cells were detached. A solution of 10% FBS in PBS was added to each plate to inhibit the trypsin action. Then, cells were harvested, and the cell number was determined after several counts of a certain volume in Neubauer hematocytometer.

### 2.5. Cell Apoptosis

Apoptotic cell nuclei were assessed by Tunnel staining using the In Situ Cell Death Detection Kit, according to standard protocol based on manufacturer's instructions (ROCHE, Cat. No. 11 684 795 910). Cell cultures were counterstained with Hoeschst 33358 (5 *μ*g/mL) which stained the nuclei of all cells. Administration of doxorubicin is known to induce apoptosis and was used as a positive control in order to certify the selected method.

### 2.6. Cell Injury

Cellular injury was assessed by LDH enzyme release in cultured medium. Culture medium was collected at the end of the experiment for the measurement of lactate dehydrogenase (LDH) activity (IU/L) using an ELISA kit (Quantichrom LDH Kit, DLDH-100, BioAssay Systems, USA). Measurements were performed with Tecan Genios system. LDH release was expressed in each group as percentage of the non-treated group.

### 2.7. Protein Isolation and Measurement of Thyroid Hormone Receptors

After washing twice with PBS, the cells were scraped into 400 *μ*L lysis buffer containing 20 mM HEPES, pH 7.9, 10 mM KCl, 1 mM EDTA, 10% glycerol, 0.2% NP-40, 0.5 mM DTT, 0.5 mM PMSF, 5 *μ*g/mL aprotinin, and 5 *μ*g/mL leupeptin. A small quantity of total lysate was kept and the remainder centrifuged at 12000 g for 1 min at 4°C. The nuclear fraction was prepared by resuspension of the pellet in buffer containing 20 mM HEPES, pH 7.9, 0.42 M NaCl, 0.2 mM EDTA, 1.5 mM MgCl_2_, 25% glycerol, 0.5 mM DTT, 0.5 mM PMSF, 5 *μ*g/mL aprotinin, and 5 *μ*g/mL leupeptin and incubated with agitation for 1 hour at 4°C before centrifugation for 10 min at 12,000 g. The resulting supernatants were collected and used for protein analysis of the nuclear fraction. Protein concentrations were determined by the BCA assay method. After boiling for 5 min (with 4% SDS, 2% mercaptoethanol, and 0.004% bromophenol blue), a quantity of 15 *μ*g protein from nuclear or total fraction was separated on 7.5% SDS-PAGE using a Bio-Rad Mini-Protean gel apparatus. For Western blotting, proteins were transferred electrophoretically to a nitrocellulose membrane (Hybond ECL) at 100 V and 4°C, for 1.5 h using Towbin buffer. After Western blotting, filters were probed with specific antibodies against TR*α*1 (Abcam Rabbit polyclonal to TR*α*1, ab53729, dilution 1 : 1000, o/n at 4°C) and TR*β*1 (Affinity Bioreagents, MA1-216, dilution 1 : 1000, o/n at 4°C). Filters were incubated with appropriate anti-mouse (Amersham) or anti-rabbit (Cell Signaling) HRP secondary antibodies. Immunoreactivity was detected by enhanced chemiluminescence using Lumiglo reagents (New England Biolabs). Chemiluminescence was detected by the image analysis system FluorChem HD2 (Alpha Innotech Corporation, 14743, Catalina Street, San Leandro, CA) equipped with a CCD camera and analysis software. Five samples from each group were loaded on the same gel. Ponceau staining was used in order to normalize slight variations in protein loading.

### 2.8. Determination of Kinase Signaling Activation

Filters were probed with specific antibodies. Membranes with total protein extracts were blocked with 5% nonfat milk in TBS-Tween for 60 min and then probed with specific antibodies against total and phospho-ERK (Cell Signalling Technology, dilution 1 : 1000) and total and phospho-Akt (Cell Signalling Technology, dilution 1 : 1000), overnight at 4°C. Filters were incubated with appropriate anti-rabbit HRP secondary antibody (Cell Signaling, 1 : 4000, 1 h R.T.), and immunoreactivity was detected by enhanced chemiluminescence using Lumiglo reagents (New England Biolabs). Immunoblots were quantified using the FluorChem HD2 system (Alpha Innotech Corporation, 14743, Catalina Street, San Leandro, CA). Data were obtained from *n* = 5 samples for each group.

### 2.9. Statistics

Values are presented as mean (S.E.M.). Data were analyzed with single factor analysis of variance ANOVA across groups. An unpaired independent sample *t*-test or nonparametric Mann-Whitney test was performed, as appropriate. A two-tailed test with a *P* value less than 0.05 was considered significant.

## 3. Results

### 3.1. Cell Morphology

**Figure 1:**
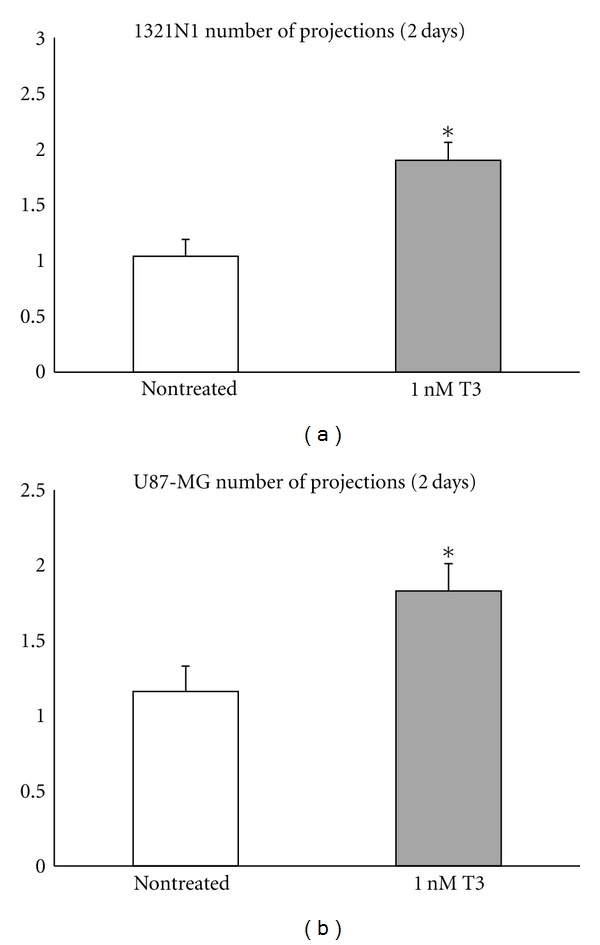
T3 induced cell re-differentiation as indicated by the significant increase in the ratio of number of projections to total cell number both in 1321N1 cells (a) and U87-MG cells (b) at 2 days. Data were derived from approximately 100 cells in each group. *P < 0.05 versus non-treated.

T3 induced cell redifferentiation in both cell lines studied as indicated by the significant increase in the number of perisomatal filopodia like neurites. Thus, in 1321N1 cells, the ratio of total number of projections to total number of cells was 1.04 (0.14) for non-treated versus 1.9 (0.11) in T3 treated, *P* < 0.05. In U87-MG cells, the ratio of total number of projections to total number of cells was 1.16 (0.14) for nontreated versus 1.83 (0.19) in T3 treated, *P* < 0.05. (Figure 1).

### 3.2. Cell Proliferation

**Figure 2:**
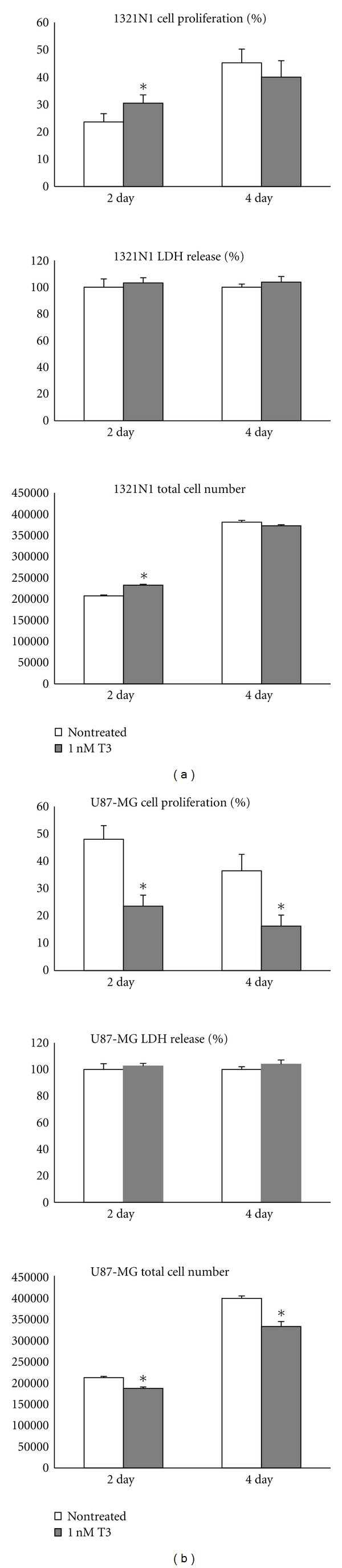
Cell proliferation index, LDH release, and total cell number in non-treated 1321N1 (a) and U87-MG (b) cells and after exposure to 1 nM T3 medium concentration for 48 h and 96 h. Cell proliferation index was assessed as the percentage of BrdU-positive nuclei to the total number of nuclei, while LDH release was expressed in each group as percentage of the untreated group. *P < 0.05 versus non treated.

In 1321N1 cell cultures, at two days, BrdU-immunostained cell nuclei were found to be 23.6% (3) in non-treated versus 30.5% (3) in T3 treated, *P* < 0.05. At 4 days, cell proliferation was shown to be 45.2% (5) in non-treated versus 40% (6) in T3 treated, *P* > 0.05 (Figure 2). 

In U87MG cell cultures, at 2 days, BrdU-immunostained cell nuclei were 48% (5) in nontreated versus 23.6% (4) in T3 treated, *P* < 0.05. In addition, after 4 days, cell proliferation was shown to be 36.5% (6) in non-treated versus 16.3% (4) in T3 treated, *P* < 0.05. (Figure 2).

### 3.3. LDH Release and Apoptosis

No change in LDH release was observed either in 1321N1 or U87MG cell cultures (Figure 2). Apoptosis was not detected either in 1321N1 or U87MG cells (data not shown).

### 3.4. Total Cell Number

In 1321N1 cell cultures, at two days, total cell number was found to be 207183 (2145) in non-treated versus 232366 (2390) in T3 treated, *P* < 0.05. At 4 days, total cell number was 381105 (4100) in non-treated versus 372433 (2595) in T3 treated, *P* > 0.05 (Figure 2).

In U87MG cell cultures, at 2 days, total cell number was found to be 211300 (2078) in non treated versus 186166 (3122) in T3 treated, *P* < 0.05. In addition, after 4 days, total cell number was 396866 (5791) in non-treated versus 331133 (11652) in T3 treated, *P* < 0.05 (Figure 2).

### 3.5. Thyroid Hormone Receptors Expression

**Figure 3:**
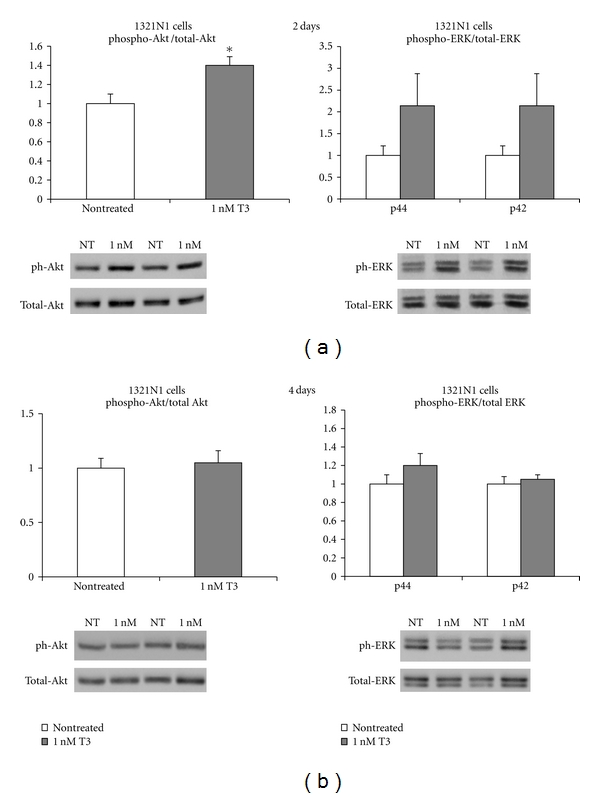
Thyroid hormone receptor *α*1 and *β*1 expression in 1321N1 cells versus U87-MG cells. Representative western blotting images are shown. P.C. corresponds to positive control. *P < 0.05 versus 1321N1 cells.

A 2.9-fold increase in the expression of TR*α*1 receptor was observed in U87MG cells as compared to 1321N1, *P* < 0.05. TR*β*1 receptor was undetectable in both cell lines (Figure 3).

### 3.6. Levels of Phospho-Akt and Phospho-ERK after T3 Treatment

**Figure 4:**
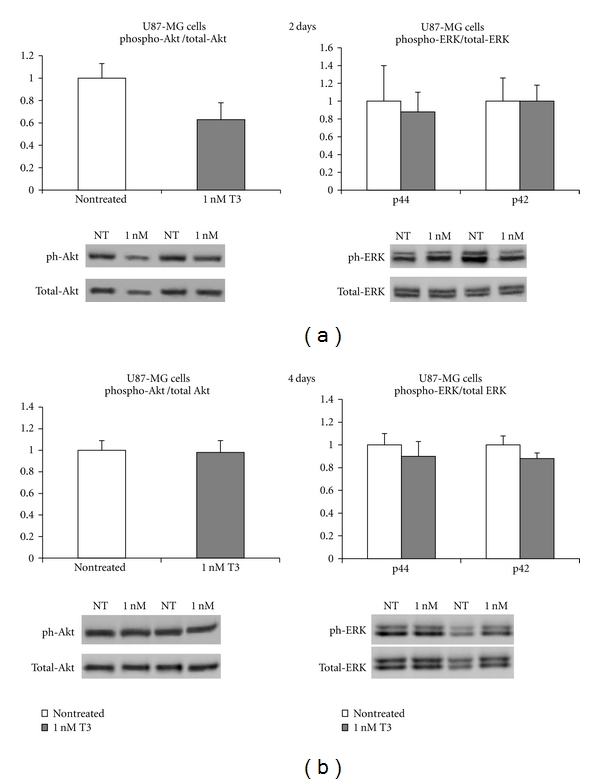
Phosphorylated levels of Akt and p44, p42 ERK after exposure of 1321N1 cells for 2 days (a) and 4 days (b) in 1 nM T3 as compared to non treated cells. Data were derived from *n* = 5 samples in each group. Representative Western blotting images are shown. *P < 0.05 versus non treated.

At two days, the ratio of p44 and p42 phospho-ERK to total ERK in 1321N1 cells was increased 2.0-fold in T3-treated cultures (*P* > 0.05) as compared to non-treated cells. Furthermore, the ratio of phospho-Akt to total Akt was found to be 1.4 higher in T3 treated cells as compared to non-treated cells, *P* < 0.05. At 4 days, no differences in the ratio of p44 and p42 phospho-ERK to total ERK and phospho-Akt to total Akt were observed between the two groups (Figure 4).

**Figure 5:**
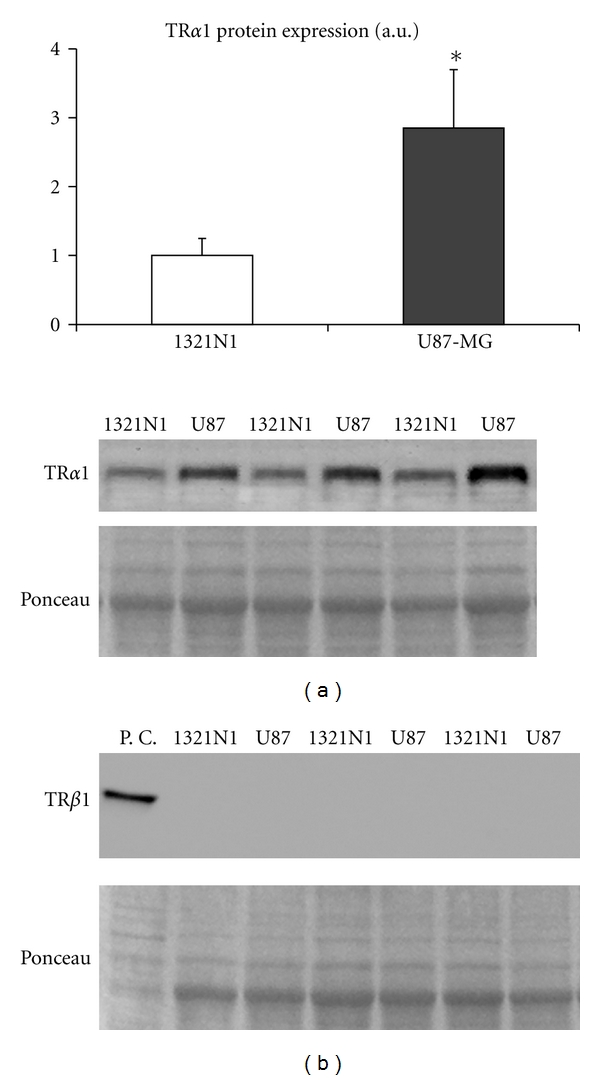
Phosphorylated levels of Akt and p44, p42 ERK after exposure of U87-MG cells for 2 days (a) and 4 days (b) in 1 nM T3 as compared to non treated cells. Data were derived from *n* = 5 samples in each group. Representative Western blotting images are shown. *P < 0.05 versus non treated, **P < 0.05 versus non treated and 1 nM T3 treated cells.

In U87MG cells, no differences in the ratio of p44 and p42 phospho-ERK to total ERK and phospho-Akt to total Akt were observed between the two groups either at 2 or 4 days (Figure 5).

## 4. Discussion

It is now recognized that TH has important regulatory actions beyond cell metabolism. TH is critical for cell differentiation, proliferation, and survival during development, and later in adult life may have regenerative/reparative action under pathological conditions [14–16]. This unique effect could potentially be of therapeutic value in cancer therapy [17]. Thus, in the present study, we explored the effects of TH treatment on cell differentiation, proliferation, and survival using two different glioma cell lines, the 1321N1, an astrocytoma grade II, and U87MG, a glioblastoma grade IV cell line. T3 was used at medium concentration of 1 nM which is in the range of near physiological concentrations and has been previously shown to suppress cell proliferation in neuroblastoma cells [5]. This treatment resulted in cell redifferentiation in both cell lines studied as indicated by the morphological changes and the marked increase in the number of perisomatal filopodia like neurites. This finding is in accordance with previous reports showing a transforming effect of T3 in neuroblastoma cells [5]. A series of genes related to neuroblastoma cell differentiation are shown to be responsive to TH [18]. It is of note that this unique effect of TH has also been shown in other cancer cells and may be of physiological and therapeutic relevance [19].

Our study further showed that the two cell lines responded differently to TH treatment as regards cell proliferation with the more aggressive tumor cells to be more sensitive. Thus, in 1321N1, T3 treatment resulted in increased cell proliferation at two days which declined thereafter, while T3 had no effect on cell injury. In contrast, in the U87MG cell line, T3 markedly suppressed cell proliferation without increasing cell injury. The potential underlying mechanisms of this cell-type-dependent action of TH on cell proliferation are not fully understood. Long-term TH effects are mediated via thyroid hormone receptors (TRs). TRs are transcription factors which regulate important genes related to cell differentiation, proliferation, and survival [17]. It is now recognized that TRs are altered in pathological conditions with important physiological consequences. Thus, we have previously shown that TRs can change in the myocardium after ischaemic stress or in cardiac cells exposed to growth stimuli [20, 21]. Similarly, there is increasing evidence that alterations in TRs are common events in cancer [4]. On the basis of this evidence, we explored whether altered TR expression in these two cell lines could possibly underlie the differential T3 effect on cell proliferation. Interestingly, TR*α*1 was found to be overexpressed in U87MG cell line compared to 1321N1, while TR*β*1 receptor was undetectable in both cell lines. This finding may indicate a potential implication of TR*α*1 receptor in T3 action on glioma cell tumors. In fact, several lines of evidence support this notion. TR*α*1 receptor has a unique dual mode of function depending on thyroid hormone availability. Thus, TR*α*1 in its unliganded state (aporeceptor) instead of being inactive exerts repressive or inducible effect on the transcription of T3 inducible or repressive genes by recruiting corepressor complexes with histone deacetylase [22]. This is of important physiological relevance during development with TR*α*1 to be overexpressed at early embryonic stages when TH is low, resulting in cell proliferation and declines thereafter with the rise of TH resulting in cell differentiation [1]. This fetal pattern of TR*α*1 expression reemerges under pathological conditions and may lead to pathological hypertrophy [20, 21] or promote cell cancer proliferation [5]. The addition of TH prevents the development of pathological cardiac hypertrophy [14] and suppresses cancer cell proliferation [5]. Taken together, these data reveal an important role of TR*α*1 in glioma cell aggressiveness and response to TH. However, this issue merits further investigation.

Our study further explored whether this differential expression of TR*α*1 had an impact on the activation of growth kinase (ERK and Akt) signaling activation induced by T3 treatment. These cascades are important regulators of cancer growth and cancer cell survival [23–25]. Both Akt and ERK are active in gliomas and have been associated with tumor aggressiveness [26–29]. Interestingly, the present study showed that T3 could significantly increase p-Akt levels in 1321N1 and not in U87MG cell line while having no effect on ERK activation in either cell line. This is in contrast with the acute nongenomic effect of T3 on U87MG cell line which was shown to involve the activation of ERK cascade [12].

In conclusion, T3 can redifferentiate glioma tumor cells. However, the T3 effect on cell proliferation appears to be dependent on the type of tumor cell line with aggressive tumors to be more sensitive to thyroid hormone treatment. TR*α*1 receptor may, at least in part, be implicated in this response.
