# Erratum to “Cell-Type-Dependent Thyroid Hormone Effects on Glioma Tumor Cell Lines”

**DOI:** 10.1155/2012/176281

**Published:** 2012-11-28

**Authors:** Alexandros Liappas, Iordanis Mourouzis, Athanasios Zisakis, Konstantinos Economou, Robert-William Lea, Constantinos Pantos

**Affiliations:** ^1^Department of Pharmacology, University of Athens, 75 Mikras Asias Avenue, Goudi, 11527 Athens, Greece; ^2^School of Pharmacy and Biomedical Sciences, University of Central Lancashire, Preston, Lancashire, PR1 2HE, UK

In the paper at doi:10.4061/2011/856050, all the authors' names and surnames are in reverse order. The authors list should be corrected as follows.

Alexandros Liappas, Iordanis Mourouzis, Athanasios Zisakis, Konstantinos Economou, Robert-William Lea, and Constantinos Pantos. 

